# A digital health program enhances walking capacity and health status in symptomatic peripheral artery disease

**DOI:** 10.1093/ehjdh/ztag072

**Published:** 2026-05-12

**Authors:** Vishal Amlani, Bartosz Dobies, Carl-Magnus Wahlgren, Manne Andersson, Birgitta Sigvant, Anders Gottsäter, Moncef Zarrouk, Olga Nilsson, Hallur Hallsson, Linda Karlsdottir, Sæmundur Oddsson, Tryggvi Thorgeirsson, Joakim Nordanstig

**Affiliations:** Institute of Medicine, Department of Molecular and Clinical Medicine, University of Gothenburg, Sweden; Department of Vascular Surgery, Sahlgrenska University Hospital, 413 45 Gothenburg, Sweden; Sidekick Health Digital Therapeutics®, Kopavogur, Iceland; Department of Vascular Surgery, Karolinska University Hospital and Karolinska Institutet, Stockholm, Sweden; Department of Biomedical and Clinical Sciences, Faculty of Health Sciences, Linköping University, Linköping, Sweden; Department of Surgery, Ryhov Hospital, Jönköping, Sweden; Region Värmland, Centre for Clinical Research and Education, Department of Surgical Sciences, Uppsala University, Uppsala, Sweden; Lund University, Department of Medicine, Skåne University Hospital, Malmö, Sweden; Vascular Centre, Department of Cardiothoracic and Vascular Surgery, Skåne University Hospital, Malmö, Sweden; Department of Vascular Surgery, Karolinska University Hospital and Karolinska Institutet, Stockholm, Sweden; Sidekick Health Digital Therapeutics®, Kopavogur, Iceland; Sidekick Health Digital Therapeutics®, Kopavogur, Iceland; Sidekick Health Digital Therapeutics®, Kopavogur, Iceland; Sidekick Health Digital Therapeutics®, Kopavogur, Iceland; Institute of Medicine, Department of Molecular and Clinical Medicine, University of Gothenburg, Sweden; Department of Vascular Surgery, Sahlgrenska University Hospital, 413 45 Gothenburg, Sweden

**Keywords:** Peripheral artery disease, Intermittent claudication, Digital health

## Abstract

**Aims:**

Intermittent claudication (IC) is the most common manifestation of peripheral artery disease (PAD), an atherosclerotic condition associated with high cardiovascular morbidity and mortality. Management targets cardiovascular risk reduction and limb function, but effective non-invasive treatments for limb symptoms remain limited. The aim of this prospective, multicentre randomized controlled trial was to evaluate whether a 12-week multimodal smartphone-based digital health programme improves walking capacity and symptoms in patients with IC.

**Methods and results:**

The IPAD trial compared standard care alone with standard care plus a 12-week digital health intervention focused on lifestyle modification and physical activity, incorporating behavioural change techniques and gamification. The primary observer-blinded endpoint was maximum walking distance (MWD) during a 6-min walk test (6MWT), contextualized against minimal clinically important difference thresholds. Secondary endpoints included pain-free 6MWT distance and health-related quality of life (HRQoL). Over 21 months, 155 patients were randomized 1:1. Mean (SD) age was 71.8 (7.6) years. Compared with controls, the intervention group showed greater improvement in MWD (+21.44 m; 95% confidence interval [CI] 6.00–36.87; *P* = 0.007), exceeding the predefined 12-m MCID at the group level, and in pain-free walking distance (+32.95 m; 95% CI 0.68–65.23; *P* = 0.045). The relative risk of achieving an individual 12-m MCID did not differ between groups (risk ratio [RR] 1.27; 95% CI 0.91–1.76; *P* = 0.162). Exploratory analysis using a 20.1-m MCID showed a higher proportion of patients achieving the MCID in the intervention group (RR 1.97; 95% CI 1.16–3.34; *P* = 0.012). HRQoL improved nominally, with a between-group benefit on the EuroQol-5 Dimensions visual analogue scale (+6.29 points; 95% CI 1.33–11.20; *P* = 0.013).

**Conclusion:**

The digital health programme resulted in a clinically meaningful improvement in walking capacity in PAD patients with IC.

## Graphical Abstract

**Graphical Abstract:**
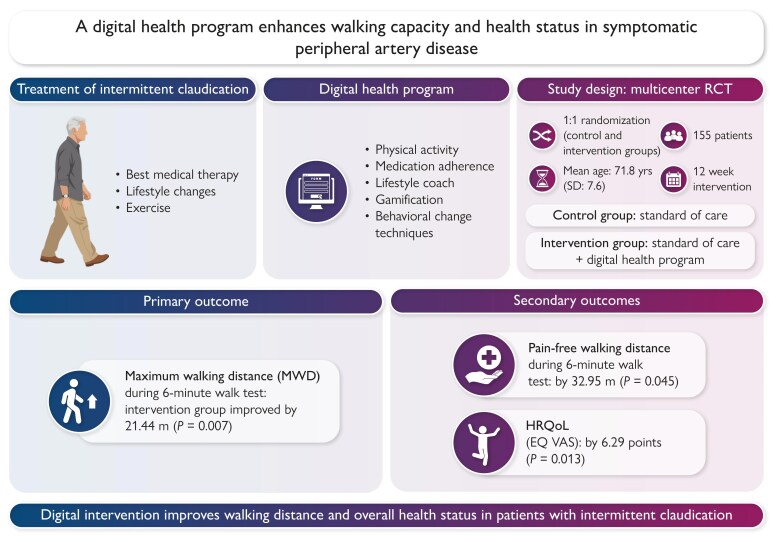

## Introduction

Peripheral artery disease (PAD) is an escalating public health concern, where the most common manifestation is intermittent claudication (IC) of vascular origin which affects approximately 30 million individuals worldwide.^1^ While IC can lead to both functional loss and impaired health-related quality of life (HRQoL), it is also associated with a markedly increased risk of major adverse cardiovascular and limb events, and cardiovascular death.^2^

A pivotal objective in the medical management of IC is to mitigate the occurrence of such undesirable events via secondary prevention strategies.^2^ These encompass utilizing cardioprotective medications alongside optimal management of dyslipidaemia, hypertension, and diabetes.^2^ Although PAD should be considered a coronary artery disease risk equivalent, PAD patients are often underserved in terms of essential cardioprotective medical therapies.^3^ Moreover, lifestyle modifications including dietary adjustments, cessation of smoking, and engagement in regular physical activity play a crucial role in PAD. Cigarette smoking is a modifiable risk factor and can lead to rapid disease progression, greater risk of complications, poor post-procedural outcomes, compromised functional status, and increased hospitalizations.^4^ Exercise therapy is recommended as an integral component of standard care as it improves IC symptoms.^5^ The effect of exercise may be further enhanced by using behaviour change techniques to promote and sustain a healthier lifestyle.^5^ Exercise therapy offers multiple benefits, including improved blood flow, enhanced muscle function, and increased cardiorespiratory fitness, all of which boost walking capacity, raise pain thresholds, and slow disease progression.^6,7^ Supervised exercise therapy (SET) is promoted in guidelines^5^ due to observed walking capacity benefits over non-supervised exercise regimens. However, patient adherence and health care availability currently limit the overall effectiveness of SET in the IC patient population, and a recent trial demonstrated both non-inferiority and cost-effectiveness of a home-based exercise programme vs. SET.^8,9^

While vascular clinicians are typically well-versed in these fundamental components of PAD management, the practical delivery of PAD care remains fragmented. These practical challenges are compounded by limited awareness and knowledge among PAD patients, inadequate access to care including support for sustainable lifestyle change, and by gaps in the care continuity between primary and secondary healthcare settings, as well as among various specialists involved in secondary care management.^10^ With PAD prevalence projected to rise sharply and healthcare costs increasing, there is a clear need for resource-efficient, patient-centred care models.^1^ We hypothesized that a PAD-specific digital health programme, using behavioural change strategies via smartphone, could streamline care by supporting lifestyle modification, exercise, and medication adherence. The primary aim was to assess whether a 12-week programme improves 6-min walk distance vs. standard care. Secondary aims included effects on HRQoL, lifestyle behaviours, and medication adherence

## Methods

### Overall study design

**Figure 1:**
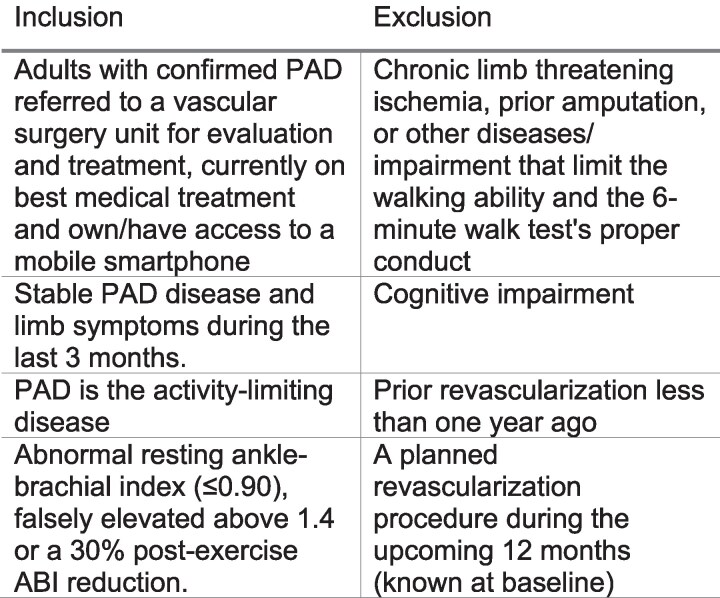
Inclusion and exclusion criteria for the IPAD trial.

The IPAD (a digital health Intervention in Peripheral Artery Disease) trial was a prospective, multicentre, two-armed, open-blinded endpoint, and randomized controlled trial. The trial was conducted across five Swedish vascular surgery units and was centrally coordinated from the Sahlgrenska University Hospital in Gothenburg. Patients were recruited among individuals with IC referred to the participating units for revascularization evaluation between October 2021 and July 2023. All patients provided written consent to the trial. The study protocol was approved by the Swedish Ethical Review Authority (entry no. Dnr 2021-02164) and was preregistered in the ClinicalTrials.gov database (NCT05029739). A detailed statistical analysis plan was compiled ahead of data base lock. Inclusion and exclusion criteria are listed in *Figure 1*.

### Baseline characteristics, randomization, and blinding

The vascular surgeon obtained the patient baseline characteristics including anthropometric measurements, educational level, smoking status (current/former/never), Rutherford class, pharmacological treatments, and comorbidities. The ABI at rest was noted from the most affected side and was considered normal if ranging between 0.9 and 1.4.^5^ After consent, patients were randomized 1:1 via a computerized system (stratified by centre and smoking status) to the intervention (digital programme + standard care) or control (standard care) group. Randomization and data entry were managed in an electronic case report form (Medicase, Medicase Inc., Sweden) by site research nurses. Patient-reported outcomes were self-completed and verified for completeness. Blinded nurses conducted pre- and post-intervention 6MWT, with patients instructed not to disclose group assignment to them throughout the study. A blinded statistician performed the analysis, with group allocation unblinded after final reporting.

### Interventions

Regardless of treatment allocation, all patients received comprehensive verbal and written information regarding the diagnosis of PAD. During this informational session, vascular surgeons communicated to all patients that lower limb revascularization was not deemed necessary. Both the verbal and written information provided underscored the significance of lifestyle modifications and informed all patients that exercise could alleviate IC symptoms and enhance walking performance. Guideline-recommended secondary prevention medications were initiated or adjusted as needed during the trial enrolment visit.^4^

Patients randomized to the digital health programme were guided by the research nurse to successfully download the Sidekick PAD smartphone application (Sidekick Health Digital Therapeutics®) onto their personal smartphones. This PAD-specific digital health programme was developed using behaviour change techniques for lifestyle change, incorporating ‘gamification’ techniques delivered through daily tasks and challenges to promote healthy lifestyle choices and increased physical activity. The application featured functionality for altruistic feedback mechanisms and provided educational content on disease management, self-care, stress and sleep management, as well as daily tasks related to nutrition and physical activity. Notably, the application included a virtual walking exercise programme. Each week, the patients received individualized feedback through a private chat function. Medication reminders were integrated into the application to enhance adherence to cardiovascular protective medications. Moreover, an optional smoking cessation programme was made available in the application. One week after randomization, smokers allocated to the digital health program were thus offered participation in this smoking cessation program directly through the application.

### Primary and secondary endpoints

#### Primary endpoint

The primary endpoint was change in 6MWT maximum walking distance from the baseline visit to the conclusion of the 12-week intervention period. This IC-modified 6MWT protocol has excellent test–retest reliability for patients with IC.^11,12^ To further contextualize the observed 6MWT changes, the primary endpoint analysis also included a prespecified comparison of the likelihood of achieving a 6MWT minimal clinically important difference (MCID) set at 12 m of improvement.^13^

#### Key secondary endpoints

Key secondary endpoints included disease-specific HRQoL measured pre- and post-intervention using the validated Swedish version of the six-item Vascular Quality of Life Questionnaire (VascuQoL-6)^14,15^ and medication adherence during the study period evaluated using the Morisky Medication Adherence Scale (MMAS-8).^16,17^

#### Secondary endpoint

The secondary endpoint involved changes in the overall quality of life and general health status from baseline to 12 weeks assessed with the EuroQol-5 Dimensions-5 Levels (EQ-5D-5L) instrument including the Visual Analogue Scale (VAS) component, which was analysed separately.

#### Exploratory endpoints

As a part of the exploratory analysis, two endpoints related to the primary endpoint were analysed. These included pain-free 6MWT walking distance and the likelihood of achieving the 20.1-m improvement in maximal 6MWT based on updated MCID estimates hypothesized to be more conservative and reflective of actual clinical improvements among the population of PAD patients in this study.^18^

Among the subgroup of patients who smoked, change in patients’ daily smoking patterns from baseline to 12 weeks was assessed using carbon monoxide breath monitors (MD Diagnostics Ltd, Kent, UK) in parallel with patient-reported smoking status (yes/no), reported daily frequency of cigarettes smoked, and the Smoking Readiness to Quit Ladder.^19^

**Figure 2:**
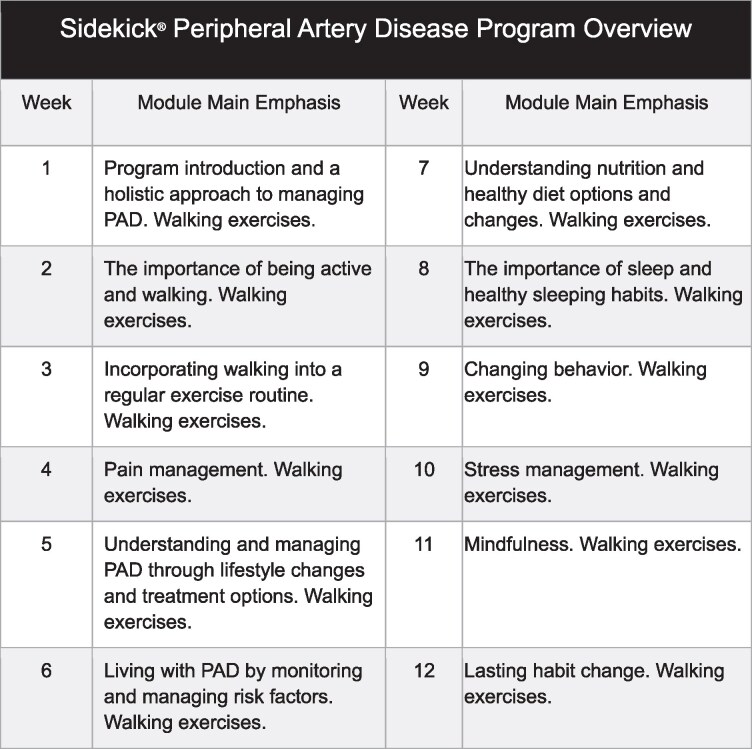
Overview of the sidekick® digital peripheral artery disease programme.

The exploratory endpoints also involved changes in the Rutherford classification assessed from baseline to the 12-week study visit. Further exploratory investigations involved in-app traffic, subgroup analyses based on health literacy scores (categorized according to Garcia-Codina),^20^ smartphone application engagement levels, and effect moderators that were revealed during analysis of the endpoints specified in *Figure 2*.

### Sample size calculation

The MCID for 6MWT was set at 12 m, reflecting the smallest change considered clinically relevant in prior literature.^21^ Assuming a pooled SD of 90 m, this corresponded to a conservative standardized effect size of *f* = 0.0665.^18,22^ Power calculations in G*Power (α=0.05, 1–β=0.80), with an assumed correlation of *r* = 0.85 between repeated measures, yielded a required sample size of 135 participants.^12,23,24^ To account for an anticipated attrition rate of 15%, the target enrolment was set at 155 patients.

## Statistical analyses

Analyses were conducted using R v2024.04.1. Two-sided tests with α=0.05 were used. Baseline characteristics were summarized descriptively. Continuous and discrete variables are reported as mean (SD) and categorical variables as *n* (%). Endpoints at baseline are shown in a separate table.

An intention-to-treat (ITT) analysis included all patients with ≥1 visit; a per-protocol (PP) analysis included patients completing all visits and actively using the app after week 1. High engagement was defined as ≥5 active days/week for ≥9 weeks; active days required ≥1 in-app mission.

Continuous outcomes were analysed using analysis of covariance (ANCOVA), and binary outcomes were analysed using logistic regression. In instances where influential outliers were detected, the ANCOVA model was refitted using robust regression (M-estimation; *rlm* function, MASS package), which down-weights outlying observations rather than removing them. This approach provides parameter estimates that are less sensitive to extreme values while retaining the same model specification. Odds ratios from logistic regression were converted to risk ratios (RRs) due to the high event rates to provide more interpretable and clinically meaningful estimates.

All models were adjusted for the same pre-specified covariates: baseline outcome value, age, sex, modified Rutherford class, ankle–brachial index (ABI), study site, and health literacy score. These covariates were selected *a priori* based on clinical relevance and previously reported prognostic value. To address multiplicity arising from multiple hypothesis testing, the Benjamini–Hochberg procedure (false discovery rate = 0.05) was applied separately to the primary and key secondary endpoints. Both raw and adjusted *P*-values are reported.

Multiple imputation (*m* = 10; R package *mice* v3.16.0) was used to address missing data. The imputation model included the same pre-specified covariates used in the analytic models (baseline outcome value, age, sex, modified Rutherford class, ABI, study site, and health literacy), supplemented with additional clinically relevant variables to improve predictive accuracy: blood pressure measurements, smoking status, educational level, civil status, and working status. The stability and convergence of the imputations were assessed visually using standard *mice* diagnostic plots (density plots comparing observed and imputed distributions and stripplots of imputed values across iterations).

Internal consistency of multi-item scales (MMAS-8, VascuQoL-6, EQ-5D-5L) was assessed using Cronbach’s α.

## Results

### Study population and baseline characteristics

**Figure 3:**
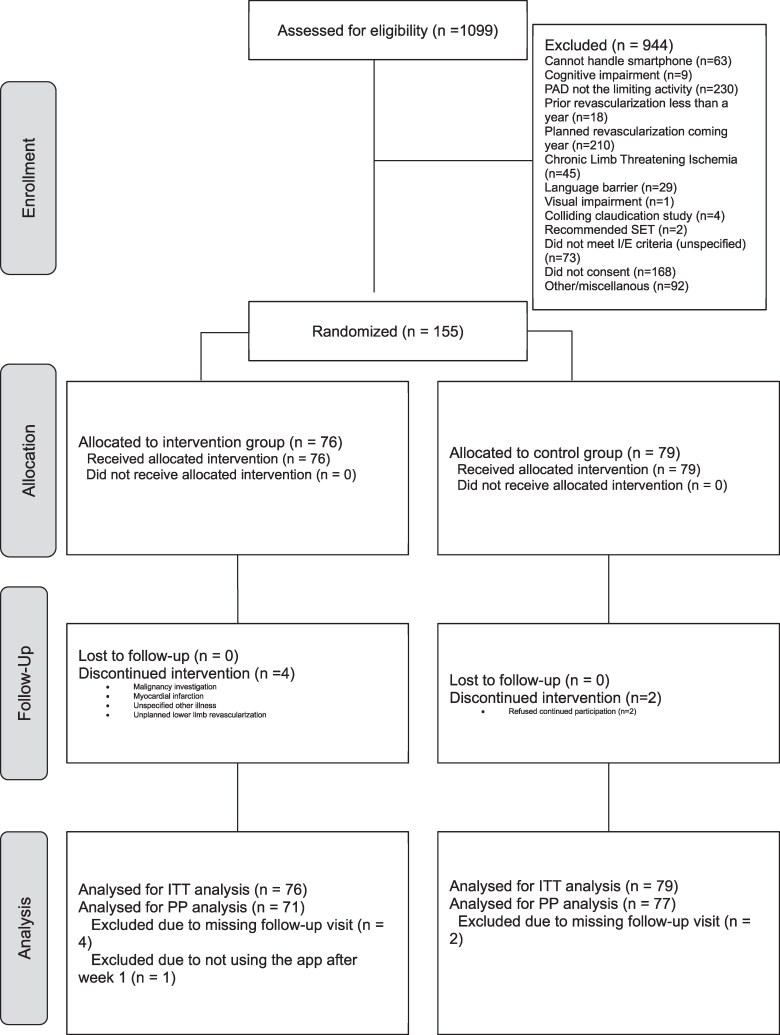
CONSORT flow diagram summarizing enrolment, treatment allocation, follow-up, and analysis of trial patients.

Overall, a total population of 1099 patients referred for IC evaluation was assessed for eligibility (*Figure 3*). Following eligibility assessment, 155 patients were enrolled in the study and randomized into either the intervention group (*n* = 76) or the control group (*n* = 79). Baseline characteristics and comorbidities are stated in *Table 1*. All randomized patients completed their baseline assessments and were included in the ITT analysis. (*Table 2*) The PP analysis excluded seven patients due to missing visits or not using the smartphone application after baseline within the intervention group.

**Table 1:** Demographic and clinical characteristics at baseline

	All patients (*n* = 155)	Intervention (*n* = 76)	Control (*n* = 79)
Sex, *n* (%)			
Female	66 (42.6)	31 (40.8)	35 (44.3)
Male	89 (57.4)	45 (59.2)	44 (55.7)
Age, years	71.8 (7.6)	71.2 (7.8)	72.4 (7.4)
BMI, kg/m2 (Missing)	26.5 (4.2)	26.6 (4.2)	26.5 (4.2)
	1	0	1
Smoking status, *n* (%)			
Current	37 (23.9)	18 (23.7)	19 (24.1)
Former (>1 month)	104 (67.1)	52 (68.4)	52 (65.8)
Never smoked	14 (9.0)	6 (7.9)	8 (10.1)
Waist circumference, cm	98.8 (12.9)	99.6 (12.9)	98.0 (12.8)
Blood pressure, mmHg			
Systolic	146.8 (18.0)	149.1 (20.4)	144.6 (15.2)
Diastolic	76.5 (10.5)	76.9 (11.6)	76.1 (9.3)
Education status, *n* (%)			
University degree	64 (41.3)	27 (35.5)	37 (46.8)
High school	59 (38.1)	28 (36.8)	31 (39.2)
Primary school	32 (20.6)	21 (27.6)	11 (13.9)
Working status, *n* (%)			
Retired	126 (81.3)	64 (84.2)	62 (78.5)
Working	25 (16.1)	11 (14.5)	14 (17.7)
Unemployed	4 (2.6)	1 (1.3)	3 (3.8)
Ankle–brachial index			
Left	0.74 (0.22)	0.76 (0.23)	0.72 (0.21)
Right	0.75 (0.23)	0.74 (0.23)	0.77 (0.23)
Lower	0.63 (0.17)	0.65 (0.19)	0.62 (0.16)
Modified Rutherford class, *n* (%)			
Mild	36 (23.2)	13 (17.1)	23 (29.1)
Moderate	74 (47.7)	40 (52.6)	34 (43.0)
Severe	45 (29.0)	23 (30.3)	22 (27.8)
Comorbidities, *n* (%)			
Diabetes (type 1 or 2)	46 (29.7)	25 (32.9)	21 (26.6)
Coronary artery disease	49 (31.6)	26 (34.2)	23 (29.1)
Chronic heart failure	13 (8.4)	8 (10.5)	8 (10.1)
Chronic kidney disease	7 (4.5)	3 (3.9)	4 (5.1)
COPD	20 (12.9)	13 (17.1)	7 (8.9)
Previous lower limb revascul.	17 (11.0)	7 (9.2)	10 (12.7)
Previous stroke or TIA	18 (11.6)	10 (13.2)	8 (10.5)
Health Literacy Score (0–16)^a^	14.2 (2.5)	14.1 (2.3)	14.3 (2.7)
Sufficient	122 (78.7)	58 (76.3)	64 (81.0)
Problematic	28 (18.1)	16 (21.1)	12 (15.2)
Inadequate	5 (3.2)	2 (2.6)	3 (3.8)
Civil status, *n* (%)			
Married	77 (49.7)	45 (59.2)	32 (40.5)
Partnership	25 (16.1)	13 (17.1)	12 (15.2)
Single	39 (25.2)	14 (18.4)	25 (31.6)
Widow/widower	14 (9.0)	4 (5.3)	10 (12.7)
Treatment and medication, *n* (%)			
Antihypertensive	134 (86.5)	66 (86.8)	68 (86.1)
Antiplatelet	139 (89.68)	68 (89.47)	71 (89.87)
Single	129 (92.81)	63 (92.65)	66 (92.96)
Dual	10 (7.19)	5 (7.35)	5 (7.04)
Anticoagulation	39 (25.16)	18 (23.68)	21 (26.58)
Full dose	16 (41.03)	8 (44.44)	8 (38.10)
Low dose	23 (58.97)	10 (55.56)	13 (61.90)
Statins^b^	146 (94.19)	73 (96.05)	73 (92.41)
High intensity	88 (60.27)	45 (61.64)	43 (58.90)
Low to moderate intensity	58 (39.73)	28 (38.36)	30 (41.10)
Other antihyperlipidemic	20 (12.90)	10 (13.16)	10 (12.66)

Results are presented using mean (SD) for continuous and discrete measures and *n* (%) for categorical;

BMI, body mass index; COPD, chronic obstructive pulmonary disease; TIA, transient ischaemic attack.

^a^Health literacy measured with HLS-EU-Q16, partly missing responses were imputed using multiple imputation method (*n* = 5) based on the adjustment covariates.

^b^Low-intensity statin ex: Simvastatin 10 mg, Pravastatin 10–20 mg; Moderate-intensity statin therapy: Atorvastatin 10–20 mg, Simvastatin 20–40 mg, Rosuvastatin 5–10 mg; High-intensity statin ex: Atorvastatin 40–80 mg 1 × 1, Rosuvastatin 20–40 mg 1 × 1.

**Table 2:** Outcome characteristics at baseline

	Intervention (*n* = 76)	Control (*n* = 79)
6MWT, m		
Maximum distance	388.7 (89.3)	395.8 (81.0)
Pain-free distance	165.8 (83.3)	191.6 (115.8)
MMAS-8 (0–8)		
Overall score	7.4 (1.0)	7.4 (0.8)
Categories	44 (57.9%)	44 (55.7%)
High	27 (35.5%)	31 (39.2%)
Moderate	5 (6.6%)	4 (5.1%)
Low		
VascuQoL-6 (0–24)	14.7 (3.2)	14.1 (3.8)
EQ-5D-5L (0–1)	0.74 (0.17)	0.68 (0.18)
EQ-VAS (0–100)	68.6 (15.7)	66.8 (15.9)
	Intervention: smokers (*n* = 18)	Control: smokers (*n* = 19)
Daily number of cigarettes smoked	9.6 (5.7)	9.5 (9.1)
Carbon Monoxide Breath Test^a^	14.7 (7.7)	16.2 (9.8)
Smoking Readiness to Quit Ladder (0–10)	6.6 (1.5)	5.8 (2.3)

Results are presented using mean (SD) for continuous and discrete measures and *n* (%) for categorical;

The numbers in brackets next to the variable name represent possible score ranges.

6MWT, six-minute walking test; MMAS-8, the eight-item Morisky Medication Adherence Scale; VascuQoL-6, the Vascular Quality of Life Questionnaire-6; EQ-5D-5L, the 5-level EQ-5D version; EQ-VAS, EQ visual analogue scale.

^a^One observation in the intervention group at baseline was removed due to measurement error.

The study patients had a mean age of 71.8 years (SD = 7.6), with 81.3% being retired. Males constituted 57.4% of the sample. Regarding smoking status, 67.1% were former smokers, and 23.9% were current smokers. The educational level was high, with 41.3% possessing a university degree and 38.1% having completed high school. The mean Health Literacy Score (HLS-EU-Q16) was 14.2 (SD = 2.5), with 78.7% of patients (*n* = 122) demonstrating sufficient health literacy, 18.1% (*n* = 28) showing problematic levels, and 3.2% (*n* = 5) having inadequate literacy. The mean (SD) ABI was 0.63 (0.17), and 47.7% of patients were categorized as having moderate disease severity (Rutherford class = 2), 29.0% as severe (Rutherford class = 3), and 23.2% as mild (Rutherford class = 1). Both intervention and control groups showed comparable baseline demographic, clinical, and outcome characteristics (*Tables 1* and *2*). For safety measures, please see Supplementary index.

### Engagement and retention with the digital health programme

**Figure 4:**
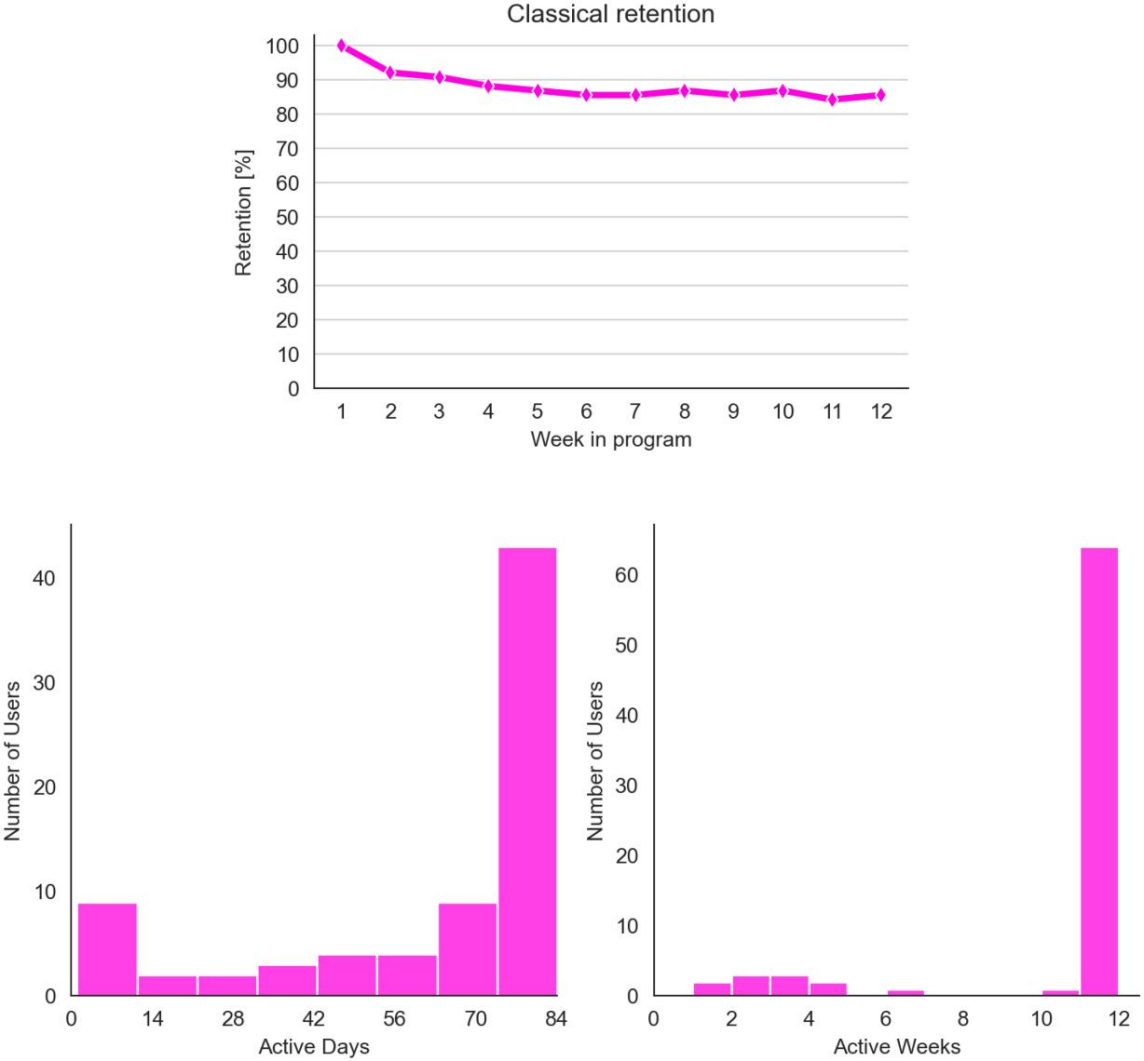
Retention and engagement of the users who were offered the digital health app. Retention represents the percentage of users retained in a particular week and engagement presents users reaching a particular number of active days and weeks. An active day/week is defined as a day/week during which the user completes at least one in-app mission.

The engagement and retention metrics indicated high participation rates with the digital health programme (*Figure 4*). Most users (86%, 65/76) remained active for over 9 weeks out of 12. This consistent engagement was reinforced by 78% (59/76) of patients staying active throughout the entire 12-week period. The average within-app task compliance was 68%, demonstrating adherence to the offered in-app activities. Furthermore, 66% (50/76) of users were highly engaged. Among 17 in-app survey respondents who declared themselves as smokers, 12 expressed a willingness to quit and were offered the additional smoking cessation module. The patients in the intervention group received a median [Q1-Q3] of 14 feedback messages [13–17], while users themselves sent a median [Q1-Q3] of 5 messages [1–8] during the 12 weeks. Approximately 49% of patients (37/76) set up medication reminders with a median medication reminder task compliance of 73%.

### Primary endpoint

**Figure 5:**
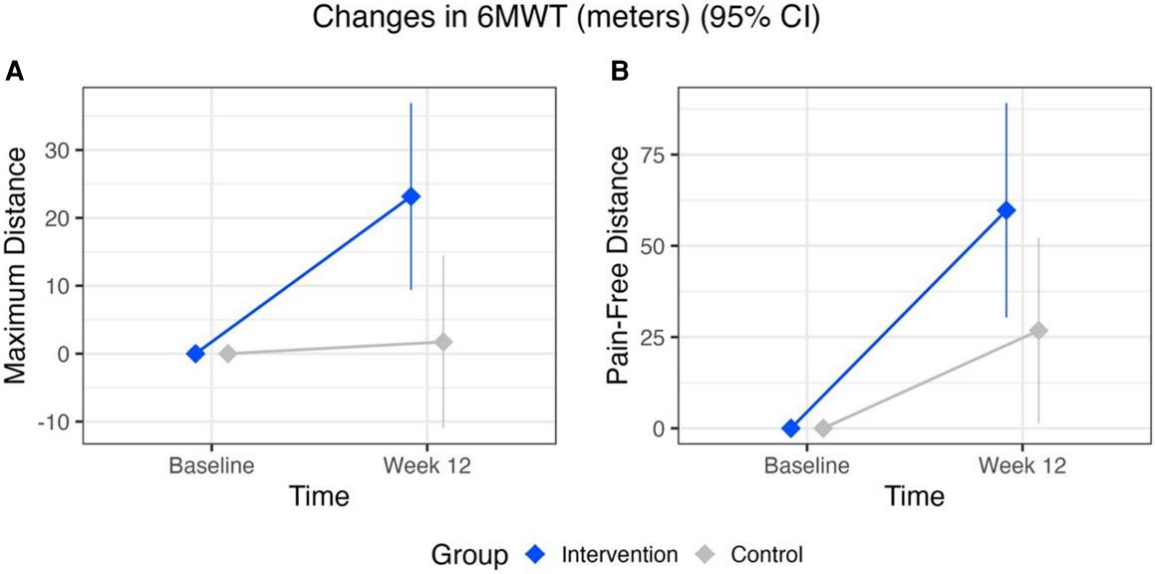
**(*A*)** changes in maximum distance walked during the 6-minute walk test from baseline to the 12-week follow-up visit, for intervention and control groups in the intention-to-treat analysis. (***B*)** Pain-free distance walked during the 6-minute walk test from baseline to the 12-week follow-up visit, for intervention and control groups in the intention-to-treat analysis.

The intervention arm demonstrated improvements in maximum 6MWT distance compared to the control group (*Table 3* and *Figure 5*). The intervention group improved on average 23.17 m (95% CI: 9.41–36.91) in ITT and 22.81 m (95% CI: 8.97–36.65) in PP analyses. In contrast, the maximum distance for the control group remained nearly unchanged, with a mean change of 1.73 m (95% CI: −10.99–14.45) in ITT and 1.48 m (95% CI: −10.72–13.69) in PP. The ITT analysis demonstrated an average between-groups difference of 21.44 m (95% CI: 6.00–36.87; *P* = 0.007) in favour of the intervention group and the PP analysis showed an average between-groups difference of 21.33 m (95% CI: 6.02–36.64; *P* = 0.006), and both remained significant after adjusting for multiple testing (*P* = 0.013).

**Table 3:** Summary of results of trial endpoints after 12 weeks in the intention-to-treat and per-protocol populations

Variable		Estimated change from baseline (95% CI)		Difference (intervention vs. control treatment) (95% CI)	*P*	*p*FDR
		Intervention	Control			
Primary endpoint						
Maximum distance in 6MWT (m)^a^	ITT	23.17 (9.41, 36.91)	1.73 (−10.99, 14.45)	21.44 (6.00, 36.87)	**0**.**007**	**0.013**
	PP	22.81 (8.97, 36.65)	1.48 (−10.72, 13.69)	21.33 (6.02, 36.64)	**0**.**006**	**0.013**
Key secondary endpoints						
MMAS-8^a^	ITT	0.30 (0.12, 0.48)	0.14 (−0.02, 0.29)	0.16 (−0.04, 0.36)	0.109	0.280
	PP	0.27 (0.09, 0.44)	0.12 (−0.04, 0.28)	0.15 (−0.05, 0.34)	0.140	0.280
VascuQoL-6	ITT	1.21 (0.25, 2.17)	0.58 (−0.29, 1.45)	0.64 (−0.45, 1.72)	0.248	0.304
	PP	1.19 (0.22, 2.16)	0.62 (−0.24, 1.48)	0.57 (−0.51, 1.65)	0.304	0.304
Secondary endpoints						
EQ-5D-5L^a^	ITT	0.056 (0.024, 0.089)	0.022 (−0.007, 0.051)	0.035 (−0.003, 0.072)	0.071	—
	PP	0.052 (0.019, 0.084)	0.019 (−0.010, 0.048)	0.033 (−0.004, 0.070)	0.081	—
EQ VAS^a^	ITT	3.55 (−0.80, 7.90)	−2.74 (−6.74, 1.25)	6.29 (1.33, 11.20)	**0**.**013**	—
	PP	3.11 (−1.33, 7.54)	−3.00 (−6.96, 0.97)	6.11 (1.19, 11.02)	**0**.**015**	—
Exploratory endpoints						
Pain-free distance in 6MWT	ITT	59.74 (30.36, 89.12)	26.78 (1.34, 52.23)	32.95 (0.68, 65.23)	**0**.**045**	—
Daily number of cigarettes smoked^a^	ITT	−0.30 (−2.33, 1.72)	0.28 (−1.53, 2.09)	−0.58 (−2.75, 1.58)	0.596	—
Carbon Monoxide Breath Test^a^	ITT	−0.79 (−6.69, 5.12)	2.31 (−2.85, 7.47)	−3.10 (−9.58, 3.38)	0.348	—
Smoking Readiness to Quit Ladder^a^	ITT	−0.43 (−1.76, 0.89)	0.17 (−0.96, 1.29)	−0.60 (−1.99, 0.79)	0.397	—

ITT, intention to treat; PP, per-protocol; CI, confidence interval; *P*, *P*-value; pFDR, *P*-value adjusted for false discovery rate; 6MWT, 6-minute walking test; MMAS-8, the eight-item Morisky Medication Adherence Scale; VascuQoL-6, the Vascular Quality of Life Questionnaire-6; EQ-5D-5L, the 5-level EQ-5D version; EQ-VAS, EQ visual analogue scale.

^a^Models for this outcome were refitted using robust regression (M-estimation) due to influential outliers.

The bolded *P*-values indicate significant results. Estimates come from models adjusted for baseline measurement, age, sex, modified Rutherford class, Ankle–Brachial Index, study centre, and health literacy score.

**Figure 6:**
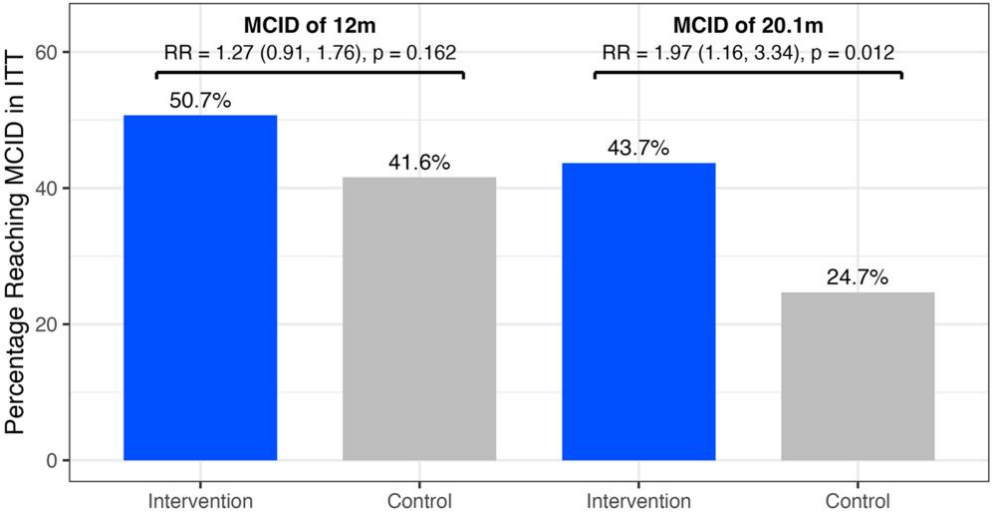
Relative number of patients in intention-to-treat reaching the minimally clinically important difference of 12 and 20.1 m with the estimated adjusted risk ratios (95% confidence interval).

To further contextualize the primary endpoint evaluation, the likelihoods of achieving an MCID of 12 m were analysed with an estimated RR of 1.27 (95% CI: 0.91–1.76; *P* = 0.162) in ITT and 1.28 (95% CI: 0.91–1.78; *P* = 0.153) in PP. However, an exploratory analysis of the likelihood of achieving a more stringent MCID of 20.1 m was significantly higher in the interventional group with an estimated RR of 1.97 (95% CI: 1.16–3.34; *P* = 0.012; *Table 3* and *Figure 6*).

Exploratory analysis on the primary endpoint indicated that females may have benefited more from the intervention than males in terms of the maximum distance walked in the 6MWT. Among male patients, the estimated difference in walking distance between the intervention and control groups was 12.70 m (95% CI: −8.11–33.51, *P* = 0.232), whereas for females, the difference was 31.49 m (95% CI: 8.35–54.64, *P* = 0.008). Overall, 24 out of 31 (77.4%) women were highly engaged with the app, compared to 26 out of 45 (57.8%) men [odds ratio 2.51 (95% CI: 0.90–7.01, *P* = 0.080)].

Other exploratory endpoints that assessed changes between groups in pain-free 6MWT distance revealed a between-group difference of 32.95 m (95% CI: 0.68–65.23; *P* = 0.045; *Table 3*, *Figure 5*), in favour of the investigational treatment arm. Additionally, comparing highly and less engaged users indicated that highly engaged patients (*n* = 50) improved their maximum 6MWT distance by an average of 26.08 m (95% CI: 3.68–48.47), while less engaged patients (*n* = 26) improved by 12.58 m (95% CI: −12.89–38.04), resulting in a non-significant difference of 13.50 m (95% CI: −11.61–38.60; *P* = 0.292, data not shown in table). For contextual reference, the estimated within-group change in the control group was 1.73 m (95% CI: −10.99–14.45), suggesting a descriptive gradient of improvement across engagement levels, while the control group showed little change.

### Medication adherence

One of the key secondary endpoints in this study was the change in medication adherence at 12 weeks, measured using the MMAS-8 scale. Although the intervention group exhibited a nominally larger improvement compared to the control group in ITT and PP, the difference was not statistically significant (*Table 3*). High levels of adherence were reported at baseline in both groups: the intervention group had a mean score of 7.38 (SD = 0.96) with 57.9% achieving the highest possible score, and the control group had a mean score of 7.44 (SD = 0.81) with 55.7% scoring at the highest level. Furthermore, the MMAS-8 scale demonstrated very low internal consistency; Cronbach’s α was 0.391 at baseline and 0.301 at week 12.

### Health-related quality of life

The other key secondary outcome was change in disease-specific health-related quality of life, measured using the validated Swedish version of the VascuQoL-6 questionnaire.^15^ Both in ITT and PP analyses, patients from the intervention group achieved nominally larger improvements, although these were not statistically significant (see *Table 3*). Exploratory analysis revealed that 16 (22.2%) patients in the intervention group reached the MCID of 3.8 points as defined by Hageman *et al*. compared to 13 (16.9%) in the control group [RR = 1.50 (95% CI: 0.71–3.14), *P* = 0.288].^9^ The internal consistency of VascuQoL-6 was high, with Cronbach’s alpha of 0.867 at baseline and 0.926 at week 12.

Furthermore, generic health status was also investigated as a secondary endpoint, using the EQ-VAS and the EQ-5D-5L scores. Changes in the visual analogue scale (EQ-VAS) yielded significant results in both ITT and PP analyses (*Table 3*). In ITT, the intervention group improved their quality of life on average by 3.55 points (95% CI: −0.80–7.90), compared to the control group, which reported a deterioration of 2.74 points (95% CI: −6.74–1.25), resulting in a difference of 6.29 points (95% CI: 1.33–11.20, *P* = 0.013). The point estimates of changes in EQ-5D-5L index scores also favoured the intervention group but did not reach statistical significance (*Table 3*). The internal consistency of the EQ-5D-5L was acceptable (Cronbach’s alpha at baseline: 0.693; at week 12: 0.762).

### Smoking cessation

This subgroup analysis focused on patients who were actively smoking at baseline and was treated as an exploratory endpoint. At 12 weeks, two additional patients identified themselves as smokers in the intervention group, with four responses missing. The number of smokers in the control group remained unchanged, with two missing responses. No differences between groups were found for the number of smokers (*P* = 0.985, not included in the table), the number of cigarettes smoked, the carbon monoxide concentration in exhaled breath, or the readiness to quit smoking (*Table 3*).

## Discussion

The main finding in this clinical trial was that the digital health programme significantly improved maximum walking distance (MWD) as measured by 6MWT after 12 weeks of therapy in patients with IC. The between-group difference of 21.44 m exceeded the predefined 12-m MCID at the group level, supporting clinical relevance of the intervention. However, responder analyses based on individual binary MCID thresholds represent a different estimand, and the relative risk of achieving the 12-m MCID did not differ between groups. An exploratory responder analysis using a larger MCID threshold of 20.1 m demonstrated a significantly higher proportion of responders in the intervention group; however, given the exploratory nature of this threshold, the result should be interpreted cautiously. In our predefined exploratory analyses, the change in pain-free 6MWT distance favoured the intervention group, with a larger effect difference observed between treatment arms for pain-free walking distance than MWD (+32.95 vs. + 21.44 m in the ITT analysis). Moreover, these observed changes in walking capacity coincided with nominally higher improvements across a broad range of patient-reported outcome metrics, which reached statistical significance for the EQ-5D-5L and EQ-VAS parameter, although the study was not powered to assess HRQoL variables. Programme engagement was high, with 84% active by week 9% and 76% completing all 12 weeks. This may reflect the generally high health literacy in our cohort (*Table 1*), which could limit external validity, as a previous study indicated that IC patients typically have low or problematic health literacy.^25^ However, the engagement could also be interpreted as a positive participant reception of the digital health programme’s content and the array of health-related activities it offered. This is noteworthy given the inherent demographic challenges in the target population, characterized by advanced age and significant comorbidity burden, which may pose obstacles to digital health programmes.^26,27^ However, we cannot exclude that implementation of this digital health programme in other PAD patient populations and routine care environments might require additional onboarding, more caregiver involvement, or hybrid care models.

The changes in MWD achieved by the digital health programme were slightly lower than what has been observed for 6MWT following SET in a recent meta-analysis. Compared with non-exercise controls, SET on average improved maximal 6MWT distances by 32.9 m (95% CI 20.6–45.6; *P* < 0.001).^28^ However, this must be considered within the different contexts of these trials and our study. Unlike prior SET studies that enrolled select, motivated IC patients, we evaluated a digital health programme targeting exercise and broader PAD self-care. Using broad inclusion criteria, we enrolled a representative IC cohort from vascular surgery clinics, with ∼30% having severe symptoms (Rutherford 3). In Sweden’s publicly funded system, the high threshold for revascularization supports inclusive recruitment for non-invasive interventions. Moreover, most previous SET studies are hampered by substantial bias known as ‘training towards the endpoint’ by utilizing the treadmills both for the exercise intervention and for the subsequent walking distance endpoint evaluation.^29^ By design, we effectively avoided such a bias due to both the nature of our intervention and due to the (blinded) 6MWT endpoint used. Also, there has been a debate about whether treadmill walking capacity (the most common endpoint metric in SET studies) adequately mirrors everyday walking ability in patients living with IC.^30,31^ Therefore, the European Society for Vascular Surgery (ESVS) clinical practice guideline on the management of asymptomatic PAD and IC advocates the use of 6MWT over treadmill testing when the purpose of the test is to determine the severity of IC.^7^ The sparse data available for SET effects on 6MWT suggest comparable effects of SET and home-based exercise regimens.^8^ Previous feasibility studies have demonstrated that telehealth-supported walking programmes in patients with peripheral arterial disease are acceptable and achievable in clinical practice.^32^ Similarly an app-based exercise therapy programme incorporating cognitive-behavioural techniques has been shown to improve walking performance and patient engagement in patients with IC.^33^ Our findings extend this evidence by providing randomized data demonstrating the effectiveness of a digital intervention on walking performance.

The ESVS guidelines recommend a stepwise IC management, starting with risk factor control, medical therapy, and exercise. In this framework, the digital programme may serve as a scalable, resource-efficient first-line option, potentially reaching more patients than traditional SET.

The study was not powered to assess all crucial PAD care components, including medication adherence and smoking cessation. Baseline MMAS-8 scores were high (∼60% at ceiling), substantially limiting the instrument’s ability to detect improvement. Although, minor, non-significant score changes suggested potential benefit, the low internal consistency of MMAS-8 further limits confidence in these findings and questions the suitability for this population.^34^ Regarding smoking cessation rates, the outcomes among the subset of active smokers at baseline (*n* = 37) were inconclusive and lacked statistical significance. Participants enrolled in the digital programme demonstrated nominal reductions in cigarette consumption and exhaled carbon monoxide levels, whereas control participants reported a marginally greater intention to quit. However, the small number of smokers included in the trial, together with the exploratory nature of this endpoint, limits the ability to draw firm conclusions. The greater benefit observed among females may reflect chance, but merits further study to inform future iterations of the Sidekick® PAD programme. Extended follow-up up to 1 year is scheduled in the trial and will further inform the long-term effects of the digital health programme and hence increase our understanding about the ability of a digital health programme to sustain healthy life choices in PAD patients.

Strengths of this study included the randomized, multicentre design, blinded endpoint assessment, and use of outcomes that are important to patients living with IC. The digital programme addressed core PAD management components and incorporated innovative features of gamification and altruistic feedback mechanisms based on behavioural science techniques. Limitations included limited power to confirm HRQoL effects and the limited number of active smokers precluded a reliable assessment of the effects of the digital smoking cessation programme. The absence of a comprehensive SET programme in the control arm represents a limitation and warrants future head-to-head comparison with the digital intervention. However, given its resource-efficient nature, the digital programme may serve as an appropriate first step within a stepwise care model for IC, with the potential to reach a substantially broader population than SET, which remains poorly available in many parts of the world.

## Conclusion

In conclusion, this comprehensive PAD-specific digital health programme, which targeted patients with mild to severe IC, improved maximal walking distance, and overall generic health status after 12 weeks of therapy.

## Clinical perspectives

Until now, there has been scarce evidence that supports the use of digital health programmes in PAD, and a recent Cochrane report highlighted a need to reliably evaluate the effects of digital health technologies on walking distance in people with IC.^35^ This study therefore adds an important new piece to the overall management puzzle of patients living with PAD and experiencing IC symptoms.

Digital health programmes with demonstrated efficacy, such as the intervention evaluated in this study, should be considered for implementation into clinical practice. One potential model is early initiation either prior to referral to a vascular outpatient clinic, or at the first outpatient visit, thereby supporting guideline-recommended exercise therapy from the outset and improving continuity of care. Within such a framework, the digital programme could complement standard care by providing continued disease education, structured exercise guidance, feedback on achieved lifestyle changes, medication adherence, and physical improvement, and monitoring between clinical encounters. Implementation at scale would likely require alignment with existing reimbursement and organizational models such as bundled care arrangements, hospital or clinic-based licensing and/or contracts with health insurers.

## Supplementary Material

ztag072_Supplementary_Data

## Data Availability

The data underlying this study and managed using an electronic case report form. Due to the sensitive nature of patient data and in accordance with applicable data protection regulations and restrictions imposed by the study’s ethical approval, the datasets are not publicly available.
