# Supplementary material for: A digital health program enhances walking capacity and health status in symptomatic peripheral artery disease
Source: Eur Heart J Digit Health. 2026 May 12;7(5):ztag072. doi: 10.1093/ehjdh/ztag072 (PMC13215472; doi:10.1093/ehjdh/ztag072)
Supplement: ztag072_Supplementary_Data [file ztag072_supplementary_data.docx]

*Supplementary Appendix*

I-PAD study

[S1. Number of study patients included in each vascular center 2](#_Toc222945586)

[S2. Results of the binary endpoints after 12 weeks for ITT and PP sample 2](#_Toc222945587)

[S3. Descriptive statistics of primary, secondary and exploratory endpoints with absolute changes after 12 weeks for the ITT sample 3](#_Toc222945588)

[S4. Safety measures & Adverse events table 3](#_Toc222945589)

[S5. List of variables 4](#_Toc222945590)

[S6. Overview of the digital health intervention structure and app interface 6](#_Toc222945591)

# **S1. Number of study patients included in each vascular center**

| **Vascular center in Sweden** | **N:** |
| --- | --- |
| Sahlgrenska University Hospital | 79 |
| Jönköping | 21 |
| Karolinska | 29 |
| Karlstad | 19 |
| Skåne | 7 |
| Total | 155 |

N: Number

# **S2. Results of the binary endpoints after 12 weeks for ITT and PP sample**

| **Variable** | | **Number of patients who achieved the MCID (%)** | | **Risk ratio,**  **vs control**  **(95% CI)** | ***p*** | ***p*FDR** |
| --- | --- | --- | --- | --- | --- | --- |
|  |  | **Intervention** | **Control** |  |  |  |
| *Primary endpoint* | | | | | | |
| Achieving the MCID of 12m in 6MWT | ITT | 36 (50.7) | 32 (41.6) | 1.27 (0.91, 1.76) | 0.162 | 0.162 |
|  | PP | 36 (51.4) | 32 (41.6) | 1.28 (0.91, 1.78) | 0.153 | 0.162 |
| *Exploratory endpoint* | | | | | | |
| Achieving the MCID of 20.1m in 6MWT | ITT | 31 (43.7) | 19 (24.7) | 1.97 (1.16, 3.34) | **0.012** | **-** |

The bolded p-value indicates a significant result. Estimates come from models adjusted for baseline measurement, age, sex, modified Rutherford class, Ankle Brachial Index, study center, and health literacy score.

Abbreviations: ITT, Intention-to-Treat; PP, Per-Protocol; CI, confidence intervals; p, p-value; pFDR, p-value adjusted for false discovery rate; 6MWT, six-minute walking test; MCID, minimal clinically important difference.

# **S3. Descriptive statistics of primary, secondary and exploratory endpoints with absolute changes after 12 weeks for the ITT sample**

| **Variable** | | **Intervention**,  n = 76 | **Control**,  n = 79 |
| --- | --- | --- | --- |
| *Primary endpoint* | | | |
| **Maximum distance in 6MWT (m)**  Baseline  Week 12 [missing, n]  Change from baseline [missing, n] | | 388.74 (89.25)  410.54 (107.37) [5]  20.31 (49.37) [5] | 395.75 (81.03)  391.00 (85.36) [2]  -6.60 (54.09) [2] |
| *Secondary endpoints* | | | |
| **MMAS-8**  Baseline  Week 12 [missing, n]  Change from baseline [missing, n] | | 7.38 (0.96)  7.62 (0.66) [4]  0.20 (0.93) [4] | 7.44 (0.81)  7.45 (0.79) [2]  0.01 (1.04) [2] |
| **VascuQoL-6**  Baseline  Week 12 [missing, n]  Change from baseline [missing, n] | | 14.66 (3.24)  15.83 (4.27) [4]  1.07 (3.29) [4] | 14.08 (3.77)  14.69 (4.33) [2]  0.66 (3.13) [2] |
| **EQ-5D-5L**  Baseline  Week 12 [missing, n]  Change from baseline [missing, n] | | 0.74 (0.17)  0.76 (0.17) [4]  0.02 (0.14) [4] | 0.68 (0.18)  0.71 (0.18) [2]  0.03 (0.16) [2] |
| **EQ VAS**  Baseline  Week 12 [missing, n]  Change from baseline [missing, n] | | 68.55 (15.72)  71.14 (17.03) [4]  2.11 (14.37) [4] | 66.84 (15.86)  64.13 (19.69) [2]  -2.82 (15.45) [2] |
| *Exploratory endpoint* | | | |
| **Pain free distance in 6MWT**  Baseline  Week 12 [missing, n]  Change from baseline [missing, n] | 165.75 (83.31)  218.31 (117.41) [5]  51.94 (109.51) [5] | | 191.58 (115.81)  211.92 (123.35) [2]  17.08 (84.27) [2] |

Note: Results are presented using mean (SD). Abbreviations: ITT, Intention-to-Treat; 6MWT, six-minute walking test; MMAS-8, the eight-item Morisky Medication Adherence Scale; VascuQoL-6, the Vascular Quality of Life Questionnaire-6; EQ-5D-5L, the 5-level EQ-5D version; EQ-VAS, EQ visual analogue scale.

# **S4. Safety measures & Adverse events table**

| **Variable** | **Intervention**,  n = 76 | **Control**,  n = 79 |
| --- | --- | --- |
| Patients with any AE, n (%) | 10 (13.6) | 4 (5.1) |
| Patients with SAE, n (%) | 9 (11.8) | 7 (8.9) |
| Total number of any AEs, n  Mild, n (%)  Moderate, n (%)  Severe, n (%)  Treatment-related, n | 11  6 (54.6)  4 (36.4)  1 (9.1)  0 | 6  2 (33.3)  4 (66.7)  0 (0)  0 |
| Total number of SAEs, n  Mild, n (%)  Moderate, n (%)  Severe, n (%)  Treatment-related, n | 9  0 (0)  5 (55.6)  4 (44.4)  0 | 9  0 (0)  4 (44.4)  5 (55.6)  0 |

AE: Adverse events, SAE: Serious adverse events. (%): percent. n: number

# **S5. List of variables**

Baseline

Randomization date

Birth date (yyy/mm/dd)

Age (yrs)

Sex (m/f)

Height (cm)

Weight (kg)

BMI (kg/m2)

Waist circumference (cm)

Ankle-Brachial Index (ABI) left side

Ankle-Brachial index (ABI) right side

Systolic blood pressure (mmHg)

Diastolic blood pressure (mmHg)

Diabetes type 1 (yes/no)

Diabetes type 2 (yes/no)

Coronary artery disease (yes/no)

Previous Myocardial Infarction (yes/no)

Chronic Heart Failure (never/former/current; if current: NYHA class I-IV)

Cardiac arrythmia (never/former/current)

Previous Stroke (yes/no)

Previous TIA (yes/no)

COPD (yes/no)

Chronic kidney disease (=eGFR <60mL/min/1.73 m2); (yes/no)

Previous Lower Limb Revascularisation (yes/no: if yes: right/left/bilateral).

Single antiplatelet therapy (yes/no)

Dual antiplatelet therapy (yes/no)

Anticoagulation therapy, fulldose (yes/no)

Anticoagulation therapy, low dose (yes/no)

Statin therapy – kolla upp

Antihypertensive treatment (yes/no)

Antidiabetics, oral (yes/no)

Antidiabetics, insulin (yes/no)

Educational level (primary school, high school, higher education)

Work status (working, unemployed, retired)

Smoking (current, former, never) – former stopped over?

Daily frequency of cigarettes smoked (smokers only)

Carbon monoxide breath monitor level (ppm) (smokers only)

Modified Rutherford class (mild/moderate/severe)* *Modified Rutherford classification: Mild claudication, Moderate claudication >200m), Severe Claudication (<200)

Most symptomatic leg (left/right/equally symptomatic)

Six-minute walking test (m)

PRO Questionnaires (see study protocol)

*Low intensity statin ex: Simvastatin 10 mg, Pravastatin 10-20 mg

Moderate Intensity statin therapy: Atorvastatin 10-20 mg, Simvastatin 20-40 mg, Rosuvastatin 5-10 mg.

High intensity statin ex: Atorvastatin 40-80 mg 1x1, Rosuvastatin 20-40 mg 1x1.

12 weeks:

Modified Rutherford class (mild/moderate/severe)* *Modified Rutherford classification: Mild claudication, Moderate claudication >200m), Severe Claudication (<200)

Six-minute walking test (m)

PRO Questionnaires (see study protocol)

Smoking (yes/no)

Carbon monoxide breath monitor value (ppm) (smokers only)

Daily frequency of cigarettes smoked (smokers only)

Weight ( kg)

BMI (kg/m2)

Waist circumference ( cm)

ABI left side

ABI right side

Systolic bp

Diastolic pb

Myocardial infarction (STEMI or NSTEMI) since baseline visit (yes/no)

PCI procedure since last visit (yes/no)

Stroke since baseline visit (yes/no)

TIA since baseline visit (yes/no)

12 months:

Modified Rutherford class

Sixminute walking test

PRO questionnaires.

Smoking (yes/no)

Daily frequency of cigarettes smoked (smokers only)

Carbon monoxide breath monito (smokers only)

Weight

BMI

Waist circumference

ABI left side

ABI right side

Systolic bp

Diastolic pb

Myocardial infarction (STEMI or NSTEMI) since baseline visit (yes/no)

PCI procedure since last visit (yes/no)

Stroke since baseline visit

TIA since baseline visit

Newly diagnosed malignancy since last visit.

## **S6. Overview of the digital health intervention structure and app interface**


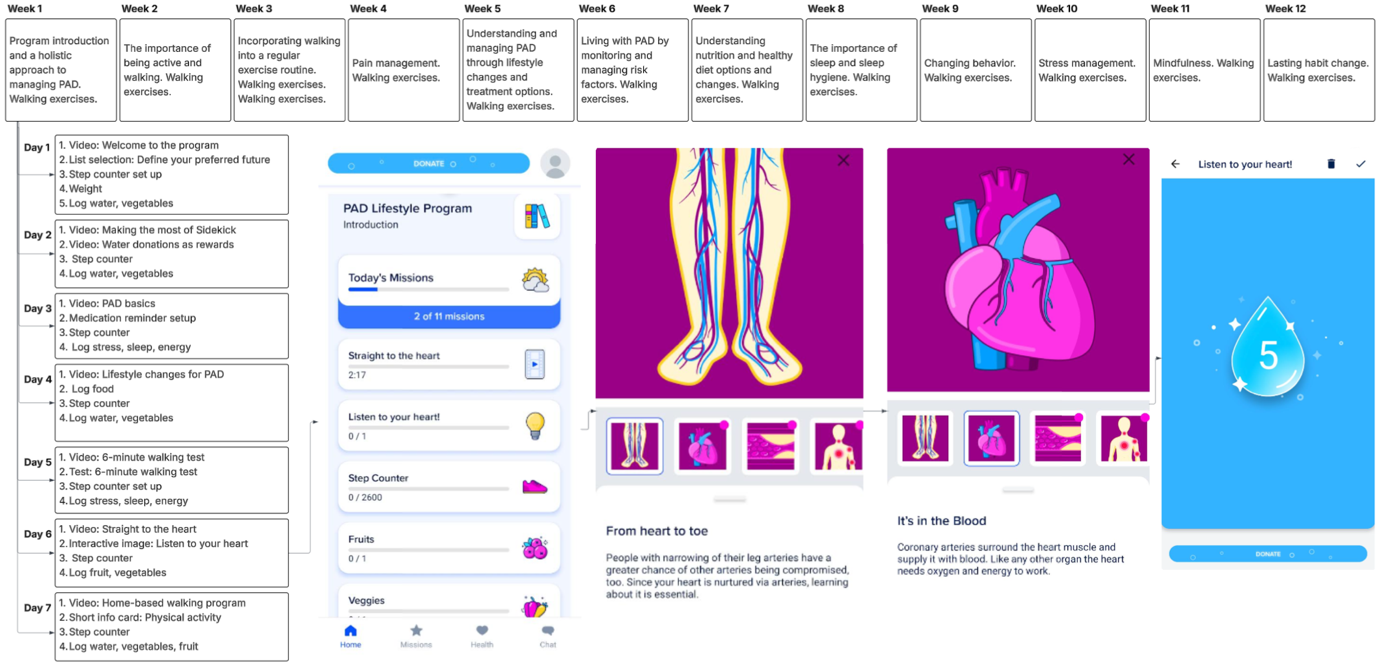


The figure provides an overview of the thematic focus of each program week, the missions delivered during Week 1, an example of the Day 6 home screen interface, and the “Listen to Your Heart” mission. Upon completion of missions, participants received water drops as a form of in-app reward. On average, participants were assigned five daily tasks throughout the duration of the program.
